# Screening of Human CYP1A2 and CYP3A4 Inhibitors from Seaweed In Silico and In Vitro

**DOI:** 10.3390/md18120603

**Published:** 2020-11-29

**Authors:** Sung-Kun Yim, Kian Kim, SangHo Chun, TaeHawn Oh, WooHuk Jung, KyooJin Jung, Chul-Ho Yun

**Affiliations:** 1 Marine Biotechnology Research Center, Jeonnam Bioindustry Foundation, 21-7, Nonggongdanji 4Gil, Wando-eup, Wando-gun, Jeollanam-do 59108, Korea; kimka3152@jbf.kr (K.K.); csh@jbf.kr (S.C.); sosoth@jbf.kr (T.O.); woohyuki@jbf.kr (W.J.); kjjung@dorip.ac.kr (K.J.); 2 School of Biological Sciences and Technology, Chonnam National University, Gwangju 61186, Korea; chyun@chonnam.ac.kr

**Keywords:** cytochrome P450, phenolic compound, carotenoid, inhibitor prediction, molecular docking

## Abstract

Phenolic compounds and carotenoids are potential inhibitors of cytochrome P450s. Sixteen known compounds, phenolic compounds and carotenoids from seaweed were examined for potential inhibitory capacity against CYP1A2 and CYP3A4 in silico and in vitro. Morin, quercetin, and fucoxanthin inhibited the enzyme activity of CYP1A2 and CYP3A4 in a dose-dependent manner. The IC_50_ values of morin, quercetin, and fucoxanthin were 41.8, 22.5, and 30.3 μM for CYP1A2 and 86.6, 16.1, and 24.4 μM for CYP3A4, respectively. Siphonaxanthin and hesperidin did not show any significant effect on CYP1A2, but they slightly inhibited CYP3A4 activity at high concentrations. In silico modeling of CYP’s binding site revealed that the potential inhibitors bound in the cavity located above the distal surface of the heme prosthetic group through the 2a or 2f channel of CYPs. This study presents an approach for quickly predicting CYP inhibitory activity and shows the potential interactions of compounds and CYPs through in silico modeling.

## 1. Introduction

Human cytochrome P450 (CYP or P450), a hemeprotein superfamily containing 57 isoforms, is responsible for the oxidation of xenobiotic chemicals including clinical drugs and environmental chemicals [1]. Five CYP isoforms (CYP1A2, 2C9, 2C19, 2D6, and 3A4) are responsible for nearly 90% of all metabolic reactions and are involved in carcinogen metabolism [2,3,4]. Among the CYPs, CYP1A2 is located predominantly in the liver and also plays an important role in the metabolism of a variety of compounds including the activation of carcinogenic aryl and heterocyclic amines [5]. CYP3A4 is the most abundant CYP enzyme present in both the liver and small intestine and is of great interest because the enzyme has been known to catalyze the metabolism of approximately 50% of therapeutic agents [6]. Therefore, a number of studies have explored the interactions of various compounds with CYPs for influence on enzymatic inhibition or activation [7,8,9].

Seaweeds are an excellent source of bioactive compounds such as polysaccharides, dietary fibers, amino acids, essential fatty acids, carotenoids, vitamins, and minerals [10,11]. Yumiko et al. [12] reported various phenolic compounds (rutin, caffeic acid, catechol, hesperidin, quercitrin, myricetin, and morin) distributed in seaweed. Fucoxanthin (FX) was extracted from brown algae and microalgae such as *Laminaria japonica*, *Eisenia bicyclis*, *Undaria pinnatifida*, *Phaeodactylum tricornutum,* and *Odontella aurita* [13,14], and siphonaxanthin was extracted from the green alga *Codium fragile* [15]. In previous studies, carotenoids have been shown to induce the expression and enzyme activity of CYPs [16,17] and to have inhibited the activity of CYPs [18,19,20]. In addition, some studies have shown that phenolic compounds inhibit the activity of CYPs [21,22].

The evaluation of drug–drug interaction is a major concern during drug research and development [23]. Some drugs act as potent enzyme inducers, whereas others are inhibitors. Since most drugs are metabolized by cytochrome P450, inhibition of CYP isoforms may cause drug–drug or food–drug interactions and may result in severe side effects [24,25]. Accordingly, the aim of this study was to investigate the inhibitory potency of various phenolic compounds and carotenoids from seaweed on human CYP1A2 and CYP3A4 as well as the enzyme kinetics of inhibition in silico and in vitro. Moreover, since the interaction of these compounds with CYPs are unknown, the present study presented the information as to how compounds access the active site using in silico modeling.

## 2. Results

### 2.1. Prediction of CYP Inhibition In Silico

The CYP1A2 and CYP3A4 inhibition tendency of various phenolic compounds and carotenoids distributed in seaweed was predicted with the DL-CYP Prediction Sever and the results were shown in Table 1. The positive controls, α-naphthoflavone (α-NF) and ketoconazole (KCZ), were correctly predicted as inhibitors of CYP1A2 [26] and CYP3A4 [27], respectively. Morin (MR) had the highest predicted value (0.75) for CYP1A2 inhibition but a moderate predicted value (0.48) for CYP3A4 inhibition. The prediction values of fucoxanthin (FX) and siphonaxanthin (SX) for CYP3A4 inhibition were 0.76 and 0.58, respectively, which were higher than other compounds. The tendency of CYP1A2 and CYP3A4 inhibition by quercetin (QT) was predicted as 0.38 and 0.36, respectively, and hesperidin (HSP) had no predicted inhibitory effect in silico.

### 2.2. Purification of Fucoxanthin and Siphonaxanthin

**Figure 1:**
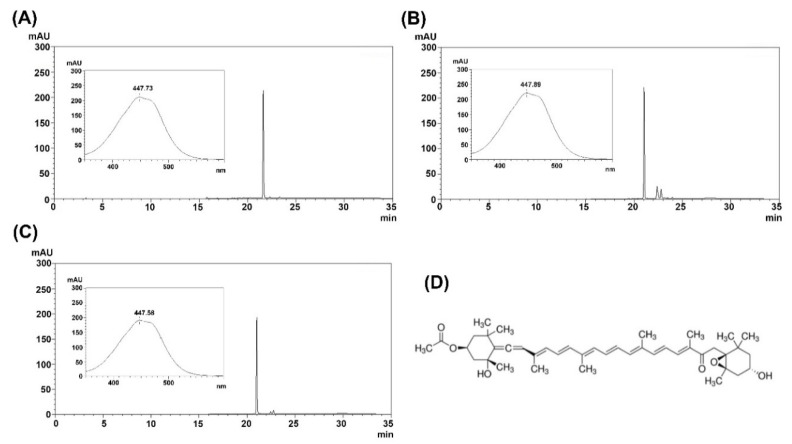
Identification and characterization of fucoxanthin. (**A**) HPLC chromatogram of standard for the detection of fucoxanthin (50 μg), (**B**) supercritical CO_2_ extract, (**C**) purified fucoxanthin, and (**D**) structure of fucoxanthin. The inset shows UV-visible spectra of the fucoxanthin peak.

**Figure 2:**
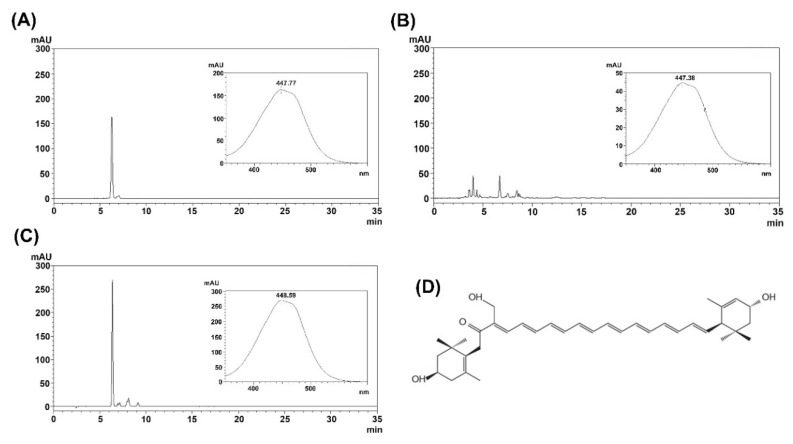
Identification and characterization of siphonaxanthin. (**A**) HPLC chromatogram of standard for the detection of siphonaxanthin (20 μg), (**B**) supercritical CO_2_ extract, (**C**) purified siphonaxanthin, and (**D**) structure of siphonaxanthin. The inset shows UV-visible spectra of the siphonaxanthin peak.

FX and SX were extracted from *U*. *pinnatifida* and *C*. *fragile*, respectively. For further purification, the supercritical CO_2_ extracts were subjected to preparative HPLC, and the purified FX and SX were analyzed by HPLC (Figure 1 and Figure 2). The purified FX and SX peaks appeared at the same retention times (21 min and 6.3 min, respectively) as their standards and their UV-visible spectra were also consistent with the standards.

### 2.3. Kinetics of Human CYP1A2 and CYP3A4 Inhibition by Test Compounds

**Figure 3:**
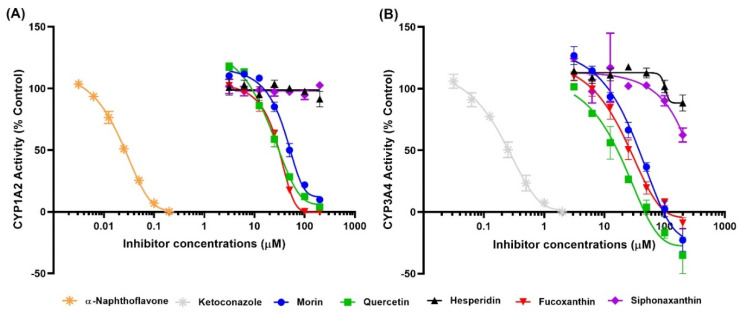
Kinetics of human CYP1A2 and CYP3A4 inhibition. The percent inhibition of test compounds against human CYP1A2 (**A**) and CYP3A4 (**B**) were plotted with Graph-Pad Prism software (Graph-Pad, San Diego, CA, USA). Each point represents the mean ± SD obtained from three independent experiments.

Inhibitory effects of the test compounds (QT, HSP, MR, FX and SX) on human recombinant CYP1A2 and CYP3A4 activity were investigated using a specific substrate (Figure 3). MR, QT, and FX inhibited the enzyme activity of CYP1A2 and CYP3A4 in a dose-dependent manner. The IC_50_ values of MR, QT, and FX were 41.8, 22.5, and 30.3 μM for CYP1A2 (Figure 3A and Table 2) and 86.6, 16.1, and 24.4 μM for CYP3A4 (Figure 3B and Table 2), respectively. SX and HSP did not show any significant effect on CYP1A2, but they slightly inhibited CYP3A4 activity in high concentrations.

### 2.4. Docking of Compounds into Human CYP1A2 and CYP3A4

**Figure 4:**
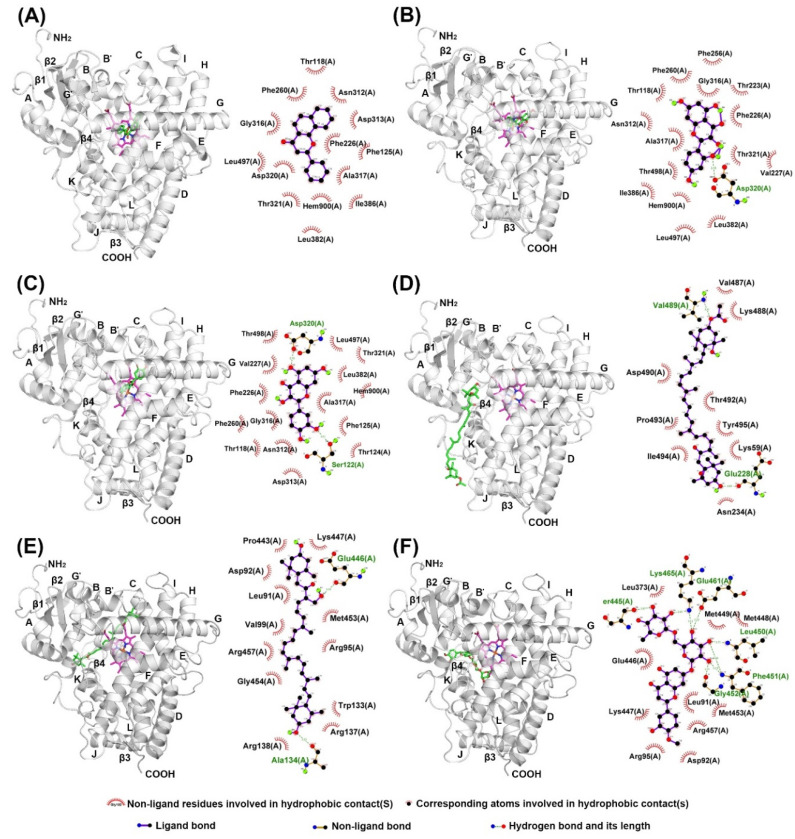
Molecular docking poses of (**A**) α-naphthoflavone, (**B**) morin, (**C**) quercetin, (**D**) fucoxanthin, (**E**) siphonaxanthin, and (**F**) hesperidin interacting with human CYP1A2 (PDB: 2HI4) analyzed with AutoDock Vina. The secondary and tertiary structures of CYP1A2 and ligand (left) and the potential protein–ligand interactions (right) were made with PyMOL and LigPlot, respectively. Ligands and heme are depicted as green and magenta sticks in 3D structures.

**Figure 5:**
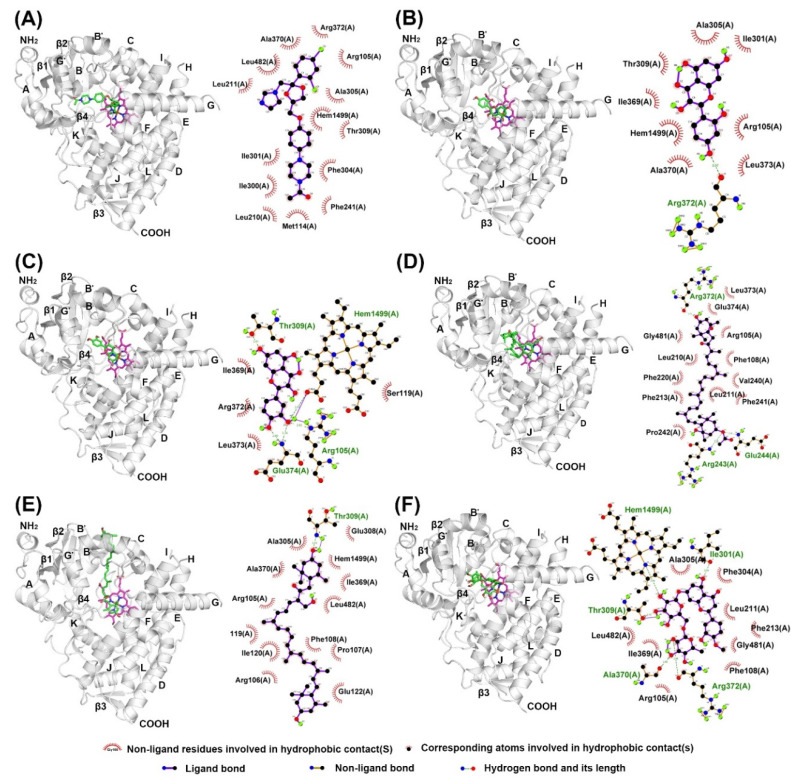
Molecular docking poses of (**A**) ketoconazole, (**B**) morin, (**C**) quercetin, (**D**) fucoxanthin, (**E**) siphonaxanthin, and (**F**) hesperidin interacting with human CYP3A4 (PDB: 2V0M, chain A) analyzed with AutoDock Vina. The secondary and tertiary structures of CYP3A4 and ligand (left) and the potential protein-ligand interactions (right) were made with PyMOL and LigPlot, respectively. Ligands and heme are depicted as green and magenta sticks in 3D structures.

**Figure 6:**
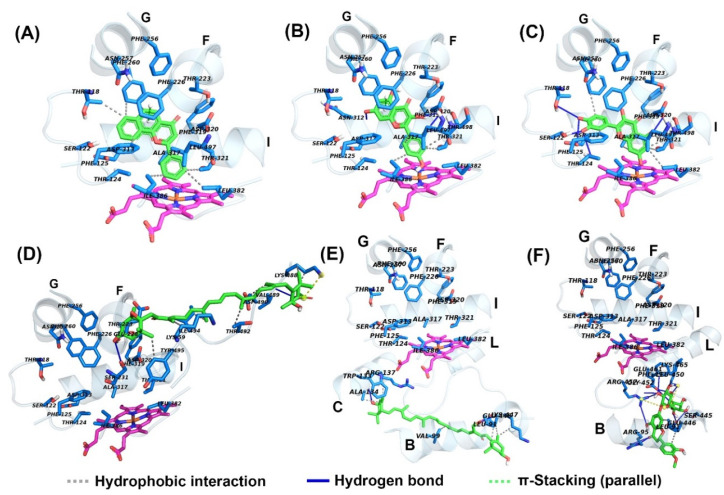
Detailed view of the binding of ligands in the human CYP1A2 (PDB: 2HI4) active site. (**A**) α-naphthoflavone, (**B**) morin, (**C**) quercetin, (**D**) fucoxanthin, (**E**) siphonaxanthin, and (**F**) hesperidin are shown in sticks, with carbon and oxygen colored green and red, respectively. The amino acid residues constituting the active site cavity are depicted in sticks, with portions of the protein backbone represented as a cartoon schematic. The heme prosthetic group is colored magenta, blue, and orange.

**Figure 7:**
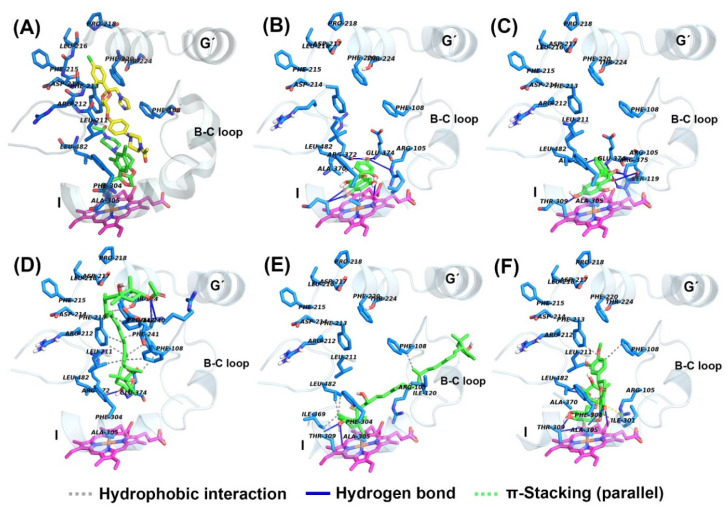
Detailed view of the binding of ligands in the human CYP3A4 (PDB: 2V0M, chain A) active site. (**A**) ketoconazole, (**B**) morin, (**C**) quercetin, (**D**) fucoxanthin, (**E**) siphonaxanthin, and (**F**) hesperidin are shown in sticks, with carbons and oxygens colored green and red, respectively. The amino acid residues constituting the active site cavity are depicted in sticks, with portions of the protein backbone represented as a cartoon schematic. The heme prosthetic group is colored magenta, blue, and orange.

The structures of α-NF and KCZ were isolated from the crystal structures of human CYP1A2 (PDB: 2HI4) and CYP3A4 (PDB: 2V0M) and were used to find the active site pocket of each CYP enzyme. After visual inspection of the top-ranked poses, the potential binding sites were found for the five compounds modeled, which were the active site cavities corresponding to the known inhibitors, α-NF and KCZ, respectively (Figure 4 and Figure 5). The binding energies and residues interacting with test compounds are presented in Table 2. The binding energy of test compounds for CYP1A2 and CYP3A4 were −4.83 ~ −7.28 kcal mol^−1^ and −6.01 ~ −8.28 kcal mol^−1^, respectively. HSP exhibited the lowest binding energy (−8.28 kcal mol^−1^) at the active site of human CYP3A4 compared to the others (Table 2). According to the analysis of docking results (Figure 4), the interactions between QT and the active site of human CYP1A2 are highly consistent with those of α-NF. In Figure 4C and Figure 6C, QT formed five hydrogen bonds (Thr118, Ser122, Thr124, Asp313, and Asp320) and seven hydrophobic interactions (Phe125, Phe260, Ala317, Thr321, Leu382, Leu497, and Thr498). Somewhat similar to α-NF, it formed 11 hydrophobic interactions (Thr118, Phe125, Phe226, Phe260, Ala317, Asp320, Thr321, Leu382, Ile386, Leu497, and Thr498), a π-cation contact (Phe226), and a water bridge (Asp320) (Figure 4A and Figure 6A). MR was also well-fitted to the active pocket of human CYP1A2, but FX, SX, and HSP interacted with amino acid residues outside the active site (Figure 4 and Figure 6). In the analysis of docking for human CYP3A4 (Figure 5), all test compounds were well-fitted into the active pocket with amino acid residues (Ala305, Thr309, and Ala370) consistent with those of KCZ (Figure 7).

## 3. Discussion

The evaluation of drug–drug interaction (DDI) is an important problem during drug research and development [28]. Some drugs act as potent enzyme inducers, whereas others are inhibitors. Since most drugs are metabolized by cytochrome P450, CYP-mediated interactions between drugs are a major category of metabolic drug–drug interactions. Therefore, CYP1A2 and CYP3A4 play an important role in assessing drug–drug interactions and avoiding adverse reactions. The early screening of CYP inhibitors has important theoretical and practical value for the development of novel drugs.

Yumiko et al. [12] reported various phenolic compounds and carotenoids distributed in seaweed. In this work, whether these compounds can inhibit human CYP1A2 and CYP3A4 activities were predicted with the DL-CYP Prediction Server in silico. Based on these results, five compounds (MR, QT, FX, SX, and HSP) were chosen, and their inhibitory effects were verified by measuring CYP1A2 and CYP3A4 activities in vitro. The inhibitory effects of test compounds against CYP enzyme activities in silico and in vitro were mostly matched, but the predicted values for FX and SX in silico were not. FX was not predicted as an inhibitor of CYP1A2 in silico. However, FX inhibited CYP1A2 and CYP3A4 enzyme activities in a dose-dependent manner with IC_50_ values of 30.2 and 24.4 μM, respectively. SX was predicted as an inhibitor of CYP3A4 in silico, but it exhibited only weak inhibitory effects in vitro.

This study confirmed that MR, QT, and FX can inhibit CYP1A2 and CYP3A4 activities in silico and in vitro. QT was the most potent inhibitor of CYP1A2 and CYP3A4 with the lowest IC_50_ values (Table 2) followed by FX and MR. This is in agreement with previous reports which have investigated the inhibitory effects of QT, FX and MR against CYP enzymes. Satomi and Nishino reported that FX inhibited the enzyme activity of CYP1A1, CYP1A2, and CYP3A4 in a dose-dependent manner with IC_50_ values of 12.5, 49.0, and 11.0 μM, respectively [17]. The inhibitory effect of FX against CYP1A2 was slightly weaker compared with CYP1A1 and CYP3A4. Elbarbry et al. [21] reported that QT did not show any significant effect on CYP1A2 or CYP2E1, but it exhibited a strong inhibitory effect against CYP2D6 and a moderate effect against CYP2C19 and CYP3A4. Interestingly, MR can inhibit CYP1A2 and CYP3A4 activities with IC_50_ values of 41.8 and 86.6 μM, respectively. These results could be obtained by screening various compounds quickly in silico with the accuracy of advanced deep learning techniques. Recently, Santes-Palacios et al. [22] reported that MR can weakly inhibit CYP1A1 with an IC_50_ value of 125.6 μM, but there are no published reports that MR inhibits other cytochrome P450 enzymes. Here, we demonstrate that deep learning techniques with improved accuracy, such as the DL-CYP Prediction Server [29], may be key tools for high-throughput screening of drugs.

In addition to the kinetics analyses, we performed molecular docking assays to explain the structural characteristics needed for inhibitory interactions. According to these results, FX, SX, and HSP are larger than α-NF, MR, and QT and could not enter into the active site cavity of CYP1A2 and interacted outside the active site of the enzyme (Figure 4 and Figure 6). Unlike SX and HSP, FX interacted with the surface around the substrate binding pockets in CYP1A2 (Figure 4D). It is thought that the activity of CYP1A2 is inhibited because FX blocks the passage through which the substrate enters. In contrast, the active site cavity of CYP3A4 is larger than that of CYP1A2, so all test compounds bound in the distal surface of the heme prosthetic group of CYP3A4 (Figure 5 and Figure 7). Sansen et al. [30] mentioned that the volume of the active site cavity in CYP1A2 was estimated to be 375 Å^3^. This is smaller than CYP3A4 and CYP2C8 with volumes of 1385 Å^3^ and 1438 Å^3^, respectively. Visible in the comparison of the active site cavities in Figure 4 and Figure 5, there is a distinct difference in that CYP1A2 is narrow and buried, while CYP3A4 is a large and open structure. Through these differences, how substrates pass through the protein to access the active site and how products egress from the active site can affect enzyme specificity and kinetics (Figure 3 and Table 2).

In molecular docking poses (Figure 4, Figure 5, Figure 6 and Figure 7), α-NF, MR, and QT bind in the cavity located above the distal surface of the heme prosthetic group through channel 2a of CYP1A2 and CYP3A4. However, FX covered the surface of channel 2f in CYP1A2 and was located in the cavity of channel 2f in CYP3A4. It is thought that the activity of CYP1A2 is inhibited because FX blocks the passage through which the substrate enters. In human CYP1A2, SX was predicted to reside on the outer surface between helix B, C and K″, and HSP to do so between helix B and K″. On the other hand, SX and HSP were located in the cavity of channel 2e of CYP3A4. So, it is observed that the small, hydrophobic ligands bind above the distal surface of the heme prosthetic group, while the large, hydrophilic ligands reside on the surface, outside of the active site cavity or inside the cavity of the alternate channel. Cojocaru et al. [31] reported on these channels between the active site and the protein surface in CYP crystal structures. Benkaidali et al. [32] reported that human CYP3A4 has four major channels (2a, 2e, 2f, and S). The channels 2a, 2f, and 2e of human CYP3A4 are enclosed by the β1 sheet, F-G and B-C block, and the C-terminal loop. The channels 2a and 2e are separated at Pro107 and Phe108 in the B-C loop, and channel 2a and 2f are separated by Thr224 in helix F′. They said that channel 2a is the biggest passage through which a ligand may pass between the F-G loop, B-C loop and β1 sheet and is the most likely route for substrate access and product egress in CYP3A4. The channel 2f is located between the F-G block and C-terminal loop and is an alternate passage to the substrate, which is close to the membrane. Channel 2a and 2f are hydrophobic. Channel 2a is bordered by SRS-1, SRS-2, and SRS-3, and channel 2f is bordered by SRS-2 and SRS-6. Channels 2a and 2e share a common part near the active site and then separate, and channel 2e and S exit in the cytosol. The residues of the mouth of channel 2e are rather polar, while those of 2a and 2f are a-polar. However, the predicted binding sites of SX and HSP, the outer surface between helix B, C, and K″ for SX and between helix B and K″ for HSP in CYP1A2, were located on the opposite side of the active site, which is predicted as the binding region of NADPH cytochrome P450 reductase. Mukherjee et al. [33] reported that the CYP interface region consists of the B, C-helix, CC′-loop, JK-loop, HI-loop, and I-helix. The NADP binding region is C′, G, J-helix, EF-loop, GH-loop, and JK-loop, and the FMN domain interface is B-helix and JK-loop. According to these results, HSP and SX were located in the FMN binding region of NADPH cytochrome P450 reductase, but they had no significant inhibitory effect on human CYP1A2.

In summary, the present data suggest that MR, QT, and FX inhibit CYP1A2 and CYP3A4 enzyme activity and that these inhibitory effects can be observed by screening various compounds quickly in silico with the accuracy of advanced deep learning techniques. In addition, controversially, the interaction of proteins and ligands could be predicted, and it could be explained how test compounds pass through proteins to access active sites, by using molecular docking assays in silico. The small, hydrophobic compounds MR and QT act as potential inhibitors of CYP1A2 and CYP3A4 in the active site cavity located above the distal surface of the heme prosthetic group through channel 2a. In addition to seaweed, there are various phenolic compounds whose efficacy is not yet known in nature. Thus, in this study, we presented an approach for high-throughput screening of the interactions of these compounds and drugs.

## 4. Materials and Methods

### 4.1. Chemicals and Reagents

Fucoxanthin (FX), morin hydrate (MR), quercetin (QT), hesperidin (HSP), siphonaxanthin (SX), ketoconazole (KCZ) and α-naphthoflavone (α-NF) were purchased from Sigma-Aldrich (St. Louis, MO, USA). Vivid™ CYP1A2 blue screening kit and Vivid™ CYP3A4 red screening kit were purchased from Thermo Fisher Scientific (Waltham, MA, USA). All analytical grade organic solvents (hexane, chloroform, acetonitrile, and methanol) used during the extraction and purification were purchased from Burdick & Jackson chemicals (Muskegon, MI, USA).

### 4.2. Seaweed Collection

The algae *Undaria pinnatifida* and *Codium fragile* were cultured in Wando, Jeollanam-do, South Korea. Sporophylls of *Undaria pinnatifida* were collected in May 2020, and *Codium fragile* was collected in August 2019. The fresh seaweed (10 kg) was washed with tap water in order to remove salt, epiphytes, and sand attached to the surface of the samples and then dried. The dried seaweeds were crushed, ground into a powder, passed through a 200 μm sieve, and then stored at −20 °C.

### 4.3. Prediction of CYP Inhibition In Silico

Various phenolic compounds (caffeic acid, catechol, dieckol, difucophlorethol A, diphlorethol, eckol, hesperidin, morin, myricetin, phloroglucinol, quercitrin, rutin, trifucol, and trifuhalol A) and carotenoids (fucoxanthin and siphonaxanthin) are found in various seaweeds. In silico, whether polyphenols and carotenoids of seaweed can inhibit human CYP1A2 and CYP3A4 activities were predicted on the DL-CYP Prediction Sever [29]. It is a free web tool to evaluate the tendency of ligands to inhibit five major CYP isoforms, namely 1A2, 2C9, 2C19, 2D6, and 3A4, based on a deep autoencoder multi-task neural network. All structure-data files (sdf) for test compounds were downloaded from PubChem (National Center for Biotechnology Information), and then these files were input to predict the tendency of inhibition against human CYP1A2 and CYP3A4, respectively. Known inhibitors against human CYP1A2 and CYP3A4, α-NF and KCZ, were used as positive controls to predict the inhibition of CYP activity. The results were expressed as values between 0 and 1.

### 4.4. Supercritical CO_2_ Extraction

FX and SX were extracted by supercritical CO_2_ from sporophylls of *U*. *pinnatifida* and *C*. *fragile* biomass, respectively. The apparatus included a high-pressure pump for CO_2_ (Eldex Laboratories, Inc., Nepa, CA, USA), a heating chamber (Reaction Engineering, Inc., Anyang-si, South Korea), a 200 mL extraction cell (Reaction Engineering, Inc., Anyang-si, Korea), and a back pressure regulator (TESCOM, St. Louis, MO, USA). The supercritical CO_2_ extraction was conducted at temperatures of 40–60 °C and pressures of 30 MPa using a semi-continuous flow-type system with CO_2_ flow rate of 2 mL min^−1^ and ethanol flow rate of 1 mL min^−1^. In each experiment, 100 g of dry seaweed powder was loaded into the 200 mL extraction vessel. The top and bottom of the extraction vessel were filled with glass beads. The extraction vessel was placed in the heating chamber to maintain the operating temperature. The extracts were collected every 1 h for 6 h, pooled together, and condensed by evaporating under reduced pressure using a rotary flash evaporator.

### 4.5. Preparative HPLC

The supercritical CO_2_ extracts were dissolved in 30 mL of n-hexane, and all the solutions were filtered using a disposable filter of 0.45 μm pore size. The 5 mL of filtered extract was then subjected to preparative HPLC (LC-6AD; Shimadzu, Kyoto, Japan) on a Cosmosile 5C_18_-AR-II (250 × 10 mm ID, Nacalai Tesque, Kyoto, Japan) column with a C_18_ guard column and eluted with an acetonitrile and water mixture at a ratio of 75:25 (*v*/*v*). The flow rate was set at 5.0 mL min^−1^ with controlled temperature at 25 °C. The DAD detector was set at a wavelength of 450 nm for FX and SX. Each FX and SX fraction were collected and condensed using a rotary evaporator. The purified samples were stored at −80 °C.

### 4.6. Analytical Methods

Purified FX and SX were analyzed by reversed phase HPLC using a LC-20AD HPLC system (Shimadzu, Kyoto, Japan) consisting of a binary pump (LC20AD XR; Shimadzu, Kyoto, Japan), an automatic injection pump (SIL-20AC XR Prominence Autosampler; Shimadzu, Kyoto, Japan), a degasser, a column oven controller, and a photodiode array detector (PDA; Shimadzu, Kyoto, Japan). The FX and SX were separated on a reverse-phase Sunfire C_18_ column (5 μm particle size, 250 × 4.6 mm ID, Waters, Milford, MA, USA) coupled to a C_18_ guard column (5 μm particle size, 15 × 4.6 mm ID), regulated at 25 °C with 20 μL sample injections. The mobile phase for FX consisted of methanol and water with a flow rate of 1 mL min^−1^. For solvent gradient conditions, methanol/water ratio was increased from 60:40 (*v/v*) to 100:0 (*v/v*) over 20 min, 100% methanol was held for next 15 min, and then methanol/water ratio was decreased from 100:0 (*v/v*) to 60:40 (*v/v*) over 25 min. To separate SX, the isocratic mobile phase was acetonitrile, methanol, and 0.1% ammonium acetate (75:15:10, *v/v/v*) at a flow rate of 1 mL min^−1^. Chromatographic peaks were identified at a wavelength of 450 nm by comparing the retention times and spectra against the known standards (Sigma-Aldrich) (St. Louis, MO, USA). Each sample was analyzed in duplicate.

### 4.7. Kinetics of Human CYP1A2 and CYP3A4 Inhibition

Human CYP1A2 and CYP3A4 activities were determined using the Vivid™ CYP1A2 blue screening kit and Vivid™ CYP3A4 red screening kit according to the manufacturer’s instructions. For the CYP1A2 and CYP3A4 inhibition assay, different concentrations (3.125–200 μM) of test compounds or inhibitors were mixed with a master pre-mix comprising of CYP450 BACULOSOMES^®^ reagent and regeneration system which contained glucose-6-phosphate and glucose-6-phosphate dehydrogenase. The mixture was incubated at room temperature for 10 min. Following incubation, each CYP enzyme-specific substrate (Vivid EOMCC for CYP1A2 and Vivid BOMR for CYP3A4) and NADP^+^ were added to start the reaction. The plate was immediately transferred into the fluorescent plate reader and read in 1 min intervals for 60 min at Ex 415 nm/Em 460 nm (CYP1A2) and Ex 550 nm/Em 590 nm (CYP3A4), respectively. The percent inhibition of test compounds or positive inhibition control were calculated using the equation:

(1) $$\%Inhibition=\left(1-\frac{X-B}{A-B}\right)\times 100\%$$

where *X* is the rate observed in the presence of test compound, *A* is the rate observed in the absence of inhibitor, and *B* is the rate observed in the presence of the positive inhibition control. The plots were made with Graph-Pad Prism software (Graph-Pad, San Diego, CA, USA).

## 4.8. Docking Studies

Chemical structures of α-NF, KCZ, MR, QT, HSP, FX, and SX were obtained from PubChem (National Center for Biotechnology Information). All test compounds in sdf format were formatted to pdbqt files with OpenBabel [34]. The three-dimensional structures for human CYP1A2 (PDB: 2HI4) [30] and CYP3A4 (PDB: 2V0M, chain A) [35] were downloaded from the Protein Data Bank [36]. The removal of counter-ions, crystallographic waters, and other ligands (except the heme group) and the addition of atomic charges and solvation parameters were done using AutoDockTools [37]. The ligands, α-NF and KCZ, were used as controls for CYP1A2 and CYP3A4, respectively. Docking calculations were carried out using AutoDock Vina [38]. Grids were centered on coordinates 4.534, 19.692, and 21.219 with 0.6 Å grid spacing and dimensions of 70 Å × 70 Å × 70 Å on *x, y*, and *z* axes for CYP1A2, and on coordinates 16.186, 6.039, and 65.714, with 0.6 Å grid spacing and dimensions of 70 Å × 70 Å × 70 Å on *x, y*, and *z* axes for CYP3A4. The top-ranked binding modes and protein-ligand interactions were visualized with PyMOL Molecular Graphics system (Shrödinger, LLC, New York, NY, USA, version 1.8), Protein-Ligand Interaction Profiler [39], and LigPlot [40].

## Figures and Tables

**Table 1:** The potential for CYP1A2 and CYP3A4 inhibition of various phenolic compounds and carotenoids distributed in seaweed, predicted with the DL-CYP Prediction Sever.

Compound Names	Predicted Values of Inhibitory Effect	
	CYP1A2	CYP3A4
α-Naphthoflavone	0.99	0.06
Ketoconazole	0.08	1.0
Caffeic acid	0.02	0.01
Catechol	0.01	0
Dieckol	0	0
Difucophlorethol A	0	0.06
Diphlorethol	0.1	0.03
Eckol	0.05	0.02
Fucoxanthin	0	0.76
Hesperidin	0	0.01
Morin	0.75	0.48
Myricetin	0.2	0.21
Phloroglucinol	0.02	0.01
Quercitrin	0	0.03
Siphonaxanthin	0	0.58
Trifucol	0.28	0.03
Trifuhalol A	0.01	0.01
Rutin	0	0
Quercetin	0.38	0.36

**Table 2:** Interaction and binding energy of ligands with human CYP1A2 and CYP3A4.

Compounds	CYP1A2			CYP3A4		
	In Vitro	In Silico		In Vitro	In Silico	
	IC_50_ (μM)	Binding Energy (kcal mol^−1^)	Interactions	IC_50_ (μM)	Binding Energy (kcal mol^−1^)	Interactions
α-Naphthoflavone (α-NF)	0.022 ± 0.003	−10.58	Thr118, Phe125, Phe226, Phe260, Ala317, Asp320, Thr321, Leu382, Ile386, Leu497, Thr498	* n.d.	n.d.	n.d
Ketoconazole (KCZ)	n.d.	n.d.	n.d.	0.21 ± 0.04	−9.39	Leu210, Ile300, Phe304, Ala305, Thr309, Ala370, Leu482
Morin (MR)	41.87 ± 2.83	−7.22	Phe226, Phe260, Asn312, Ala317, Asp320, Thr321, Ile386, Leu497, Thr498	86.6 ± 9.56	−6.01	Arg105, Ile301, Ala305, Thr309, Ala370, Arg372, Glu374
Quercetin (QT)	22.47 ± 3.32	−7.28	Thr118, Ser122, Thr124, Phe125, Phe260, Asp313, Ala317, Asp320, Thr321, Leu382, Leu497, Thr498	16.09 ± 7.46	−7.57	Arg105, Ser119, Thr309, Arg372, Glu374, Arg375
Fucoxanthin (FX)	30.26 ± 3.45	−4.83	Lys59, Glu228, Ser231, Lys488, Val489, Aps490, Thr492, Ile494, Tyr495	24.41 ± 7.42	−7.69	Phe108, Leu211, Phe213, Phe220, Val240, Phe241, Pro242, Arg243, Glu244, Arg372, Glu374
Siphonaxanthin (SX)	** n.i.	−5.97	Lys59, Glu228, Leu236, Asn247, Ala249, Val489, Asp490, Leu491, Thr492, Ile494	n.i.	−6.10	Arg105, Phe108, Ile120, Ala305, Thr309, Ile369, Leu482
Hesperidin (HSP)	n.i.	−7.30	Val54, Gly58, Lys59, Asn60, Glu228, Ser231, Gly233, Asn234, Tyr495	n.i.	−8.28	Arg105, Phe108, Leu211, Phe213, Ile301, Phe304, Ala305, Thr309, Ala370

* n.d.: no data; ** n.i.: no inhibition.

## Abbreviations

CYP	Cytochrome P450
α-NF	α-naphthoflavone
KCZ	Ketoconazole
MR	Morin hydrate
QT	Quercetin
HSP	Hesperidin
FX	Fucoxanthin
SX	Siphonaxanthin
NADP	Nicotinamide adenine dinucleotide phosphate
